# Redox-inactive ions control the redox-activity of molecular vanadium oxides†

**DOI:** 10.1039/d0sc01401j

**Published:** 2020-04-06

**Authors:** Simon Greiner, Benjamin Schwarz, Mark Ringenberg, Maximilian Dürr, Ivana Ivanovic-Burmazovic, Maximilian Fichtner, Montaha Anjass, Carsten Streb

**Affiliations:** Institute of Inorganic Chemistry I, Ulm University Albert-Einstein-Allee 11 89081 Ulm Germany carsten.streb@uni-ulm.de; Helmholtz Institute Ulm for Electrochemical Energy Storage (HIU) Helmholtzstr. 11 89081 Ulm Germany; Institute of Inorganic Chemistry I, University of Stuttgart Pfaffenwaldring 55 70569 Stuttgart Germany; Department of Chemistry and Pharmacy, Friedrich-Alexander-University Erlangen-Nuremberg Nikolaus-Fiebiger-Str. 10 91058 Erlangen Germany; Karlsruhe Institute of Technology (KIT), Institute of Nanotechnology P.O. Box 3640 76021 Karlsruhe Germany

## Abstract

Polyoxometalates are key materials for energy conversion and storage due to their unique chemical tunability and electrochemical reactivity. Herein, we report that functionalization of molecular vanadium oxides, polyoxovanadates, with redox-inert Ca^2+^ cations leads to a significant increase in their electron storage capabilities. The electrochemical performance of the Ca^2+^-functionalized dodecavanadate [Ca_2_V_12_O_32_Cl(DMF)_3_]^2−^ (=**{Ca**_**2**_**V**_**12**_**}**) was thus compared with that of the precursor compound (H_2_NMe_2_)_2_[V_12_O_32_Cl]^3−^ (=**{V**_**12**_**}**). **{Ca**_**2**_**V**_**12**_**}** can store up to five electrons per cluster, while **{V**_**12**_**}** only shows one reversible redox transition. In initial studies, we demonstrated that **{Ca**_**2**_**V**_**12**_**}** can be used as an active material in lithium-ion cathodes. Our results show how redox-inert cations can be used as structural and electrostatic stabilizers, leading to major changes in the redox-chemistry of polyoxovanadates.

## Abstract 2

The enhanced redox-activity of a molecular vanadium oxide cluster upon functionalization with redox-inert Ca^2+^ ions is reported together with initial insights into its performance as a lithium ion battery cathode.

## Graphical abstract

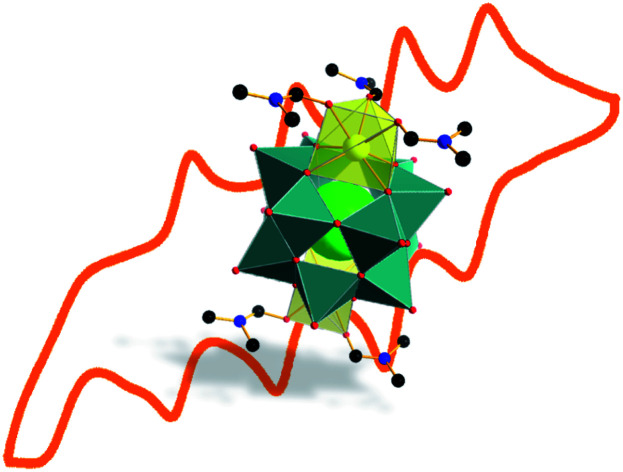

## Introduction

The growing demand for sustainable energy conversion and storage technologies has fuelled research into new materials which combine high performance, high stability and sustainability. One key materials class used in batteries, fuel cells and electrolysis is metal oxides, whose properties for (proton-coupled) electron transfer and storage can be tuned by structural or chemical modification.^1^ To-date, the controlled, predictable synthesis and rational tuning of these compounds have been a major challenge. Thus, model systems are urgently required which allow us to rationalize structure–activity relationships as well as enable their study under technologically relevant conditions, *e.g.* in battery electrodes or water electrolyzers.^2^

In this regard, molecular metal oxides, so-called polyoxometalates (POMs), have emerged as ideal molecular analogues of solid-state metal oxides.^3^ POMs are anionic metal oxo clusters of high-valent, early transition metals (often V, Mo, and W), which form by self-assembly in solution.^4^ POMs have attracted widespread interest in various technologies including energy conversion and storage,^5–7^ water electrolysis,^8–10^ redox catalysis^11–13^ and molecular electronics.^14,15^ Many of these applications are based on the ability of POMs to undergo multiple (often proton-coupled) redox-processes, a property which was initially studied mainly for tungstates and molybdates.^16^ Ground-breaking studies reported the targeted manipulation of the redox capabilities of POMs by modification of the internal POM-template, introduction of heterometals into the cluster shell, or by change of the counter-cations and/or the electrolyte.^16–18^

In contrast to these pioneering studies on Mo- and W-POMs, polyoxovanadates^19–22^ have only recently become a focal point for electrochemical reactivity tuning.^23–25^ Systematic studies have provided critical insights into the effects of heterometal-functionalization,^22,26,27^ organofunctionalization^28^ and counter-cation interactions^29,30^ on the electrochemical properties of POVs. This fundamental understanding also enabled POV application in water oxidation catalysis,^31,32^ redox-flow batteries,^33^ and lithium ion batteries,^34–36^ and as model catalyst surfaces.^37^

**Fig. 1:**
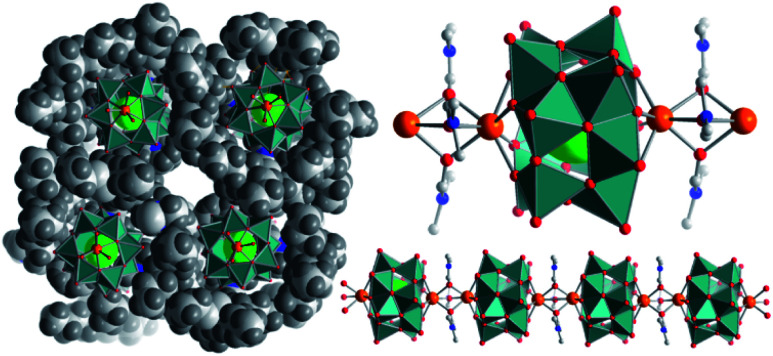
Illustration of the calcium vanadate cluster chains. Left: view along the chain propagation direction (*a*-axis), showing the separation of neighbouring chains by bulky *n*Bu_4_N^+^ cations. Right: bottom: illustration of a **{Ca**_**2**_**V**_**12**_**}** chain; top: representation of an individual **{Ca**_**2**_**V**_**12**_**}** cluster. V atoms and [VO_5_] polyhedra: teal, O: red, Cl: large green, N: blue, C: grey, and Ca: orange.

In earlier work, we have targeted the development of a class of model vanadates in which systematic variation of the heterometal enables insights into tuning possibilities of the vanadate redox-chemistry: using a so-called placeholder-cation approach,^23^ we were able to gain access to a family of transition-metal-functionalized dodecavanadate clusters, (H_2_NMe_2_)_2_[V_12_O_32_Cl]^3−^ (=**{V**_**12**_**}**),^23^ where two metal binding sites (Fig. 1) are blocked by dimethyl ammonium placeholder cations. Cation exchange is possible so that one^23,38,39^ or both sites^27,40,41^ can be functionalized with s-, d- or f-block metal cations. This enables the tuning of magnetic, redox, catalytic and photocatalytic properties.^23,27,38–41^ Thus far, the emerging redox-activity of functionalized **{V**_**12**_**}** species has been assigned to the transition metal incorporated into the cluster shell (*e.g.* Fe and Mn).^23,27,42^ Here, we hypothesized that new redox-chemistry could become possible even when large, redox-inert cations such as Ca^2+^ are employed. This is based on our recent observation that Sr^2+^-ions can coordinate to the binding sites of **{V**_**12**_**}** and enable the structural stabilization and aggregation of the cluster in the solid state and in solution.^43^ Building on these findings, we now report the structural and electrochemical consequences of Ca^2+^ coordination to **{V**_**12**_**}**. We demonstrate that structural stabilization of **{V**_**12**_**}** by redox-inactive alkali earth metal cations (Ca^2+^) leads to a dramatic enhancement of the cluster redox-activity and a significant increase in the number of accessible redox-states so that reversible storage of up to five electrons per cluster unit is possible. Initial experimental analyses provide first insights into the changes of the electronic structure upon Ca^2+^ incorporation. As a proof of concept, we demonstrate the use of this “electron sponge” as a cathode active material in Li-ion batteries.

## Results and discussion

### Synthesis and structural analysis

The di-calcium-functionalized dodecavanadate; (*n*Bu_4_N)_2_[Ca_2_V_12_O_32_Cl(DMF)_3_]·DMF (=**{Ca**_**2**_**V**_**12**_**}**, DMF = *N*,*N*-dimethyl formamide) was synthesized by reaction of (*n*Bu_4_N)_3_[H_3_V_10_O_28_] with CaCl_2_·6H_2_O in DMF and isolated as single-crystals by diffusion of acetone into the reaction solution (yield: 28% based on V). Single-crystal X-ray diffraction shows that in the lattice, individual **{Ca**_**2**_**V**_**12**_**}** clusters are linked into virtually infinite 1D chains aligned in a co-parallel fashion along the crystallographic *a* axis (Fig. 1). Linkage of neighbouring clusters occurs *via* the Ca^2+^ ions, which are coordinated to four bridging μ^2^-oxo ligands of one **{V**_**12**_**}** (*d*_Ca–O_ = 2.34–2.36 Å); in addition, each Ca^2+^ ion features three μ^2^-bridging DMF ligands (*d*_Ca–O(DMF)_ = 2.35–2.43 Å) which link to the next Ca^2+^ ion, see Fig. 1.§§Crystallographic data for: C_44_H_96_Ca_2_ClN_6_O_36_V_12_, *M*_w_ = 2012.15 g mol^−1^, monoclinic, space group *P*2_1_/*c*, *a* = 10.6954(5) Å, *b* = 29.2218(14) Å, and *c* = 28.2691(15) Å, *β* = 95.3952(16), *V* = 8796.0(8) Å^3^, *Z* = 4, *μ*(Mo Kα) = 1.433 cm^−1^, 204 760 reflections collected, 9416 unique which were used in all calculations; structure solution and refinement as done using OLEX2.^58^ Final *R*1 = 0.0815 and w*R*2 = 0.1883 (all data).

This results in a distorted, mono-capped trigonal prismatic coordination environment. In the crystal lattice, neighbouring chains are separated by charge-balancing *n*Bu_4_N^+^ cations and solvent DMF molecules. A similar chain structure of dodecavanadate clusters connected *via* solvent-coordinated Sr^2+^-cations has been recently reported by our group.^43^ Thermogravimetric analysis (TGA) in the 25–350 °C range shows the loss of four DMF molecules followed by the loss of two *n*Bu_4_N cations, which supports the assigned sum formula (see the ESI, Fig. S13†). Furthermore, ICP-OES analysis revealed a Ca : V atomic ratio of 1 : 5.98, confirming the expected stoichiometry within the **{Ca**_**2**_**V**_**12**_**}** cluster.

### Vanadium oxidation states

Charge balance considerations indicate that the **{Ca**_**2**_**V**_**12**_**}** unit has two negative charges, suggesting the presence of one reduced V^IV^(d^1^) centre. This is supported by UV-Vis-NIR studies (inset Fig. 3) which show a broad, characteristic V^IV/V^ intervalence charge transfer (IVCT) transition in the near-IR region.^44^ X-ray photoelectron spectroscopy (XPS) also supports this finding as indicated by a characteristic line broadening and the presence of a shoulder in the V2p_3/2_ region (ESI, Fig. S2†).^45^ Also, XPS analyses give O1s/V2p_3/2_ binding energy differences of 12.8 eV (assigned to V^V^) and 14.2 (assigned to V^IV^), which is in agreement with the literature values.^46,47^ Further, this finding is in line with earlier studies of **{V**_**12**_**}**, which showed that other di-metal functionalized species, such as **{Mn**_**2**_**V**_**12**_**}** (=(*n*Bu_4_N)_4_[Mn_2_^III^Cl_2_V_12_O_32_Cl]), are also accessible only upon 1-electron-reduction of the vanadate framework.^27^ Electron paramagnetic resonance (EPR) spectroscopy of **{Ca**_**2**_**V**_**12**_**}** (in DMF at 77 K) gave a broad signal with Landé-factors of *g* = 2.039 (*ca.* 330 mT) and *g* = 4.58 (*ca.* 148 mT) (see the ESI, Fig. S3†). The observed EPR signals and the lack of ^51^V hyperfine structure are in line with the presence of delocalized V^IV^ in the square pyramidal oxo environment.^48^

### Electrospray ionization mass spectrometry

Recent results on structurally closely related di-Sr-functionalized dodecavanadate clusters showed the presence of chain fragments even in dilute solutions.^43^ Therefore, we investigated the solution and gas-phase stability as well as the assembly- and disassembly mechanisms of **{Ca**_**2**_**V**_**12**_**}** by high resolution electrospray ionization mass-spectrometry (HR-ESI-MS) of a *ca.* 5 × 10^−5^ M DMF solution of **{Ca**_**2**_**V**_**12**_**}**. Analysis of the data using *m*/*z* and isotopic pattern assignments shows the parent cluster anion [Ca_2_V^IV^V^V^_11_O_32_Cl]^2−^ at *m*/*z* = 619.030 (calc.: 619.029). In addition, dimeric fragments including DMF ligands, *e.g.* {(*n*Bu_4_N)[Ca_2_V^IV^V^V^_11_O_32_Cl]_2_(DMF)}^3−^ (*m*/*z* = 930.486, calc. = 930.486) are observed, suggesting that oligomeric species related to the solid-state **{Ca**_**2**_**V**_**12**_**}** chains (Fig. 1) are present in the dissolved sample (see the ESI, Fig. S4†).

### Electrochemical and spectro-electrochemical analysis

**Fig. 2:**
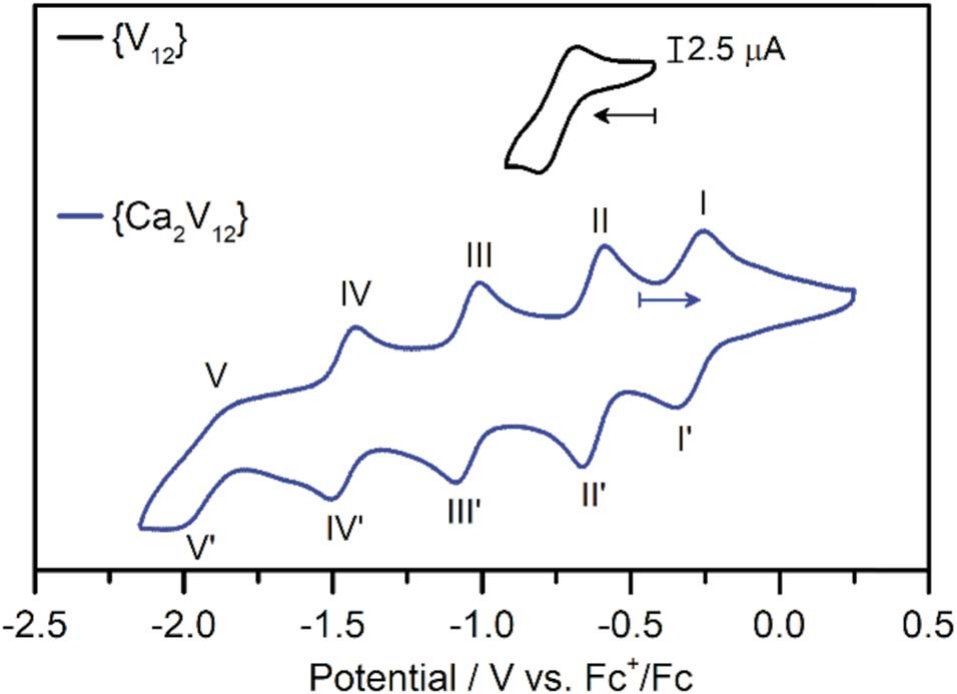
Cyclic voltammograms of **{Ca**_**2**_**V**_**12**_**}** (blue) and **{V**_**12**_**}** (black). Conditions: anhydrous, deoxygenated DMF containing 0.1 M (*n*Bu_4_N)PF_6_ as the supporting electrolyte (scan rate: 0.1 V s^−1^), [cluster]: 1 mM.

Electrochemical studies were then performed to gain insights into the effects of calcium coordination on the electronic structure and electrochemical properties of **{Ca**_**2**_**V**_**12**_**}**. The cyclic voltammogram of **{Ca**_**2**_**V**_**12**_**}** obtained in de-aerated, anhydrous DMF (containing 0.1 M (*n*Bu_4_N)PF_6_ as the electrolyte, all data referenced against Fc^+^/Fc) shows five quasi-reversible redox processes (see Fig. 2 and Table 1). The transitions were identified as one-electron processes by bulk electrolysis (see the ESI, Table S4†) and their reversibility was studied by scan-rate-dependent CV analysis, see below.

**Table 1:** Oxidation and reduction potentials of the redox processes of **{Ca**_**2**_**V**_**12**_**}**

Peak	*E*_ox_/V *vs.* Fc^+^/Fc	*E*_red_/V *vs.* Fc^+^/Fc	*E*_m_ /V *vs.* Fc^+^/Fc	Peak separation/mV
I/I′	−0.27	−0.35	−0.31	78
II/II′	−0.60	−0.68	−0.64	73
III/III′	−1.02	−1.10	−1.06	78
IV/IV′	−1.44	−1.52	−1.48	85
V/V′	−1.86	−2.05	−1.96	193

This behaviour is in striking contrast to that of the non-functionalized parent compound **{V**_**12**_**}**, which – in the same potential window – showed only one quasi-reversible one-electron redox transition at −0.74 V *vs.* Fc^+^/Fc (see the ESI, Fig. S5†).^38^ This observation emphasises the influence of a non-redox active heteroatom on the redox-activity of the cluster. This is also in contrast to earlier studies, where additional redox processes of transition-metal-functionalized dodecavanadates were assigned to the redox active heterometal.^27,38^

In order to assess the reversibility of the observed redox events for **{Ca**_**2**_**V**_**12**_**}**, we performed scan-rate-dependent CV analysis (scan-rate 50 mV s^−1^ to 2000 mV s^−1^, see the ESI, Fig. S6†). The most negative redox-transition (V/V′, *E*_1/2_ = −1.95 V) is not fully reversible based on the large peak separation and low oxidative peak current. It was therefore not further explored. Processes I/I′ to IV/IV′ show a linear relationship between the peak current *vs.* square-root of the scan rate, indicating that these four transitions are reversible, diffusion-controlled processes.^49^ However, the re-oxidation of the most cathodic signal (*E*_1/2_ = −1.48 V) deviates slightly from linearity, suggesting that the reduction is not fully reversible (see the ESI, Fig. S7–S10†). The first four processes in the CV of **{Ca**_**2**_**V**_**12**_**}** show anodic to cathodic peak height ratios near unity.

**Fig. 3:**
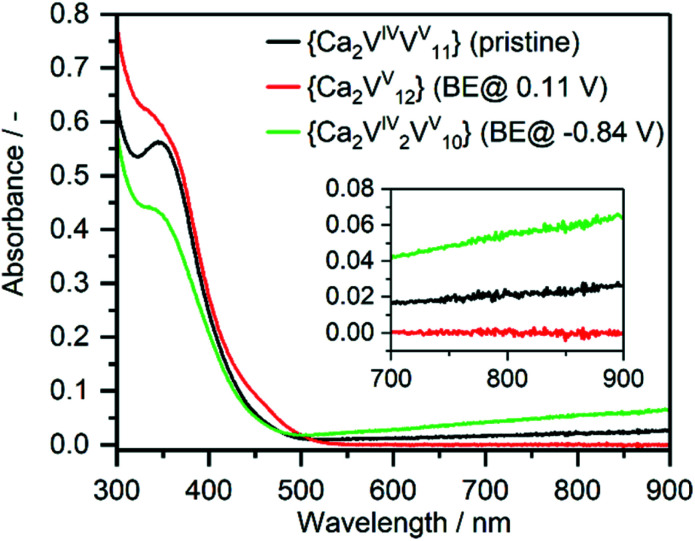
UV-Vis-NIR absorption spectrum of **{Ca**_**2**_**V**_**12**_**}** (black), after bulk electrolysis to the fully oxidized form (red) and after two-electron reduction (green).

To further explore the charging/discharging of **{Ca**_**2**_**V**_**12**_**}** and the corresponding changes in the electronic structure, we used UV-Vis-NIR/bulk-electrolysis (BE) spectro-electrochemistry. At a potential of *E* = 0.11 V, BE shows that **{Ca**_**2**_**V**_**12**_**}** is oxidized by one electron, giving the fully oxidized vanadate framework [Ca_2_V^V^_12_O_32_Cl]^−^, whereby the IVCT transition in the Vis-NIR region disappears and the solution colour changes from green to yellow (see Fig. 3). Simultaneously, the ligand-to-metal charge transfer (LMCT) transitions in the UV region increase. Similar observations have also been made during spectro-electrochemical investigations of the related iron-functionalized dodecavanadate.^38^

The re-reduction to [Ca_2_V^IV^V^V^_11_O_32_Cl]^2−^ (*E* = −0.39 V) is accompanied by a colour change back to green and a recovery of the initial IVCT transition as well as a decrease in LMCT transition. Subsequent BE at *E* = −0.84 V leads to two-electron-reduced species [Ca_2_V^V^_2_V^V^_10_O_32_Cl]^3−^ and an increase in the IVCT transition as well as a decrease in the LMCT transition; the solution colour changes to dark green. Re-oxidation (*E* = −0.34 V) of **{Ca**_**2**_**V**_**12**_**}** recovers the absorption properties of the initial one-electron-reduced compound [Ca_2_V^IV^V^V^_11_O_32_Cl]^2−^ and highlights that **{Ca**_**2**_**V**_**12**_**}** might be suitable as an electron reservoir in Li-ion battery cathodes.

Previous work had demonstrated that the presence of different cations can lead to significant shifts of the redox-processes in polyoxometalates.^29,50^ To explore this behaviour for **{Ca**_**2**_**V**_**12**_**}**, we performed cyclic voltammetry using de-aerated, anhydrous DMF containing LiPF_6_ (0.1 M) as the electrolyte (see the ESI, Fig. S11†). CV analysis showed that redox processes I/I′, II/II′ and III/III′ show only minor shifts (<100 mV). In contrast, processes IV/IV′ and V/V′ show notable shifts to more positive potentials and start to merge into one broad signal. For reduction V, the shift is most pronounced with a positive potential shift by approximately 600 mV. Also note that the re-oxidation process V′ is not observed in these measurements, which indicates either the irreversibility of this process or the merging of the oxidative processes V′ and IV′.

### Proof of concept: {Ca_2_V_12_} as a potential battery electrode material

To assess the performance of **{Ca**_**2**_**V**_**12**_**}** as a cathode active material in a prototype lithium-ion battery, electrodes containing **{Ca**_**2**_**V**_**12**_**}** (45 wt%), carbon black (40 wt%) and poly(vinylidene difluoride) (PVDF) (15 wt%) were fabricated using a standard slurry coating/doctor blading procedure described in the ESI.†^51^ In recent work, we reported that vanadates are often unstable under typical battery electrode fabrication conditions.^51^ To this end, we carefully assessed the thermal stability of **{Ca**_**2**_**V**_**12**_**}**. Thus, **{Ca**_**2**_**V**_**12**_**}** was heated under typical electrode preparation conditions (12 h, 120 °C, vacuum). The resulting product was analyzed by thermogravimetry (TGA), FT-infrared spectroscopy (FT-IR) and powder X-ray diffraction (pXRD). In sum, all data show no significant changes of the compound before and after heating, suggesting that the structure of **{Ca**_**2**_**V**_**12**_**}** is retained. This is also in line with powder X-ray diffractometry of the as-prepared electrodes which show weak but characteristic diffraction signals (due to the sample dilution by carbon black and PVDF) corresponding to the **{Ca**_**2**_**V**_**12**_**}** crystal lattice (see the ESI Fig. S15†).

**Fig. 4:**
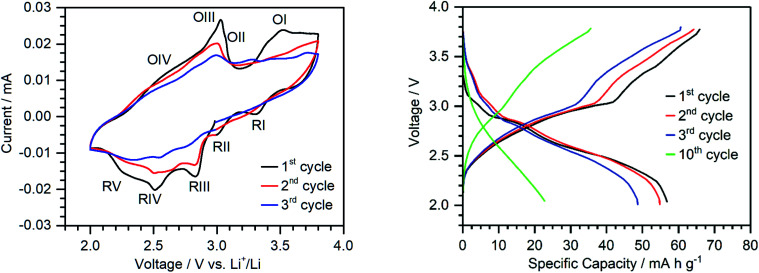
Left: cyclic voltammograms of **{Ca**_**2**_**V**_**12**_**}**-based electrodes in the range of 2.0–3.8 V *vs.* Li^+^/Li at a scan rate of 0.2 mV s^−1^. Right: voltage profile of **{Ca**_**2**_**V**_**12**_**}** half-cells cycled between 2.0 and 3.8 V *vs.* Li^+^/Li with a constant current density of 50 mA g^−1^ at 25 °C.

In order to investigate the redox processes relevant to the cathode material, cyclic voltammetry was carried out in a voltage range between 2.0 and 3.8 V *vs.* Li^+^/Li (see Fig. 4). The first CV cycle shows four distinct reduction signals at 3.30 V, 3.00 V, 2.82 V and 2.50 V (RI-RIV) with a shoulder centred at around 2.26 V (RV). The oxidation peaks are rather broad and are observed at *ca.* 3.03 V and 3.52 V. Although only two main oxidation peaks (OI and OIII) can be identified, the broadness of the peaks and the presence of shoulders (OIV and OII) suggest that these peaks correspond to multi-electron transfer reactions.

The peak shape of oxidation at around 3.03 V further suggests a possible stripping process upon oxidation.^49^ However, since a similar peak shape is observed for the cyclic voltammogram in solution in the presence of Li^+^-ions (oxidation IV), further studies are needed to verify whether the peak is due to dissociation of the ion pair upon oxidation or stripping of the cluster from the electrode. Future studies will therefore investigate the oxidation state dependent reactivity of this compound in more detail. During cycling, we note that the peak intensity is reduced and the peaks broaden further. This could be indicative of morphological changes, *e.g.* amorphization under operation (see the ESI, Fig. S15†).^52^ This is also in line with the proposed partial dissolution of **{Ca**_**2**_**V**_**12**_**}** into the battery electrolyte.^53^ Note that the peaks remain visible and no new peaks are formed, indicating no further structural changes of the cluster during cycling.

Galvanostatic charge/discharge testing using a half-cell with a metallic Li anode (in a voltage range between 2.0 and 3.8 V *vs.* Li^+^/Li at a current density of 50 mA g^−1^) delivered an initial discharge capacity of 56 mA h g^−1^ (see Fig. 4). The plateaus match the reduction peaks in the CV.

Following the equation:*Q* = (*nF*)/(3.6*M*_w_) = (*n* × 26 800)/*M*_w_where *Q* is the reversible charge/discharge capacity, *n* is the number of electrons transferred and *M*_w_ is the molecular weight, the translocation of one Li-ion/e^−^ contributes a capacity of *ca.* 13.3 mA h g^−1^. Therefore, approx. 4 vanadium atoms per **{Ca**_**2**_**V**_**12**_**}** are reduced during galvanostatic cycling in the given voltage range (theoretical capacity: 53 mA h g^−1^).

For comparison with the non-functionalized parent compound, **{V**_**12**_**}**-based electrodes were prepared following the processes described above. The **{V**_**12**_**}**-based electrodes show a significantly lower specific capacity (**{V**_**12**_**}**: *ca.* 26 mA h g^−1^; **{Ca**_**2**_**V**_**12**_**}**: 56 mA h g^−1^, voltage range 2.0–3.8 V *vs.* Li^+^/Li), providing further evidence for the higher electron storage capability of **{Ca**_**2**_**V**_**12**_**}** compared with **{V**_**12**_**}** (see the ESI, Fig. S17†). Additionally, the observed plateaus are less pronounced for **{V**_**12**_**}**, which leads to a lower average voltage and lower energy density.

Scanning electron microscope SEM) analysis of the cathode material shows the presence of large (>10 μm) **{Ca**_**2**_**V**_**12**_**}** particles. This is expected to negatively affect the battery performance due to the longer Li^+^-ion diffusion pathways.^52^ Additionally, low electronic conductivity has been reported in the literature to be a challenge for POMs in battery electrodes.^25,54^ While intrinsic conductivity has been recently reported in metal-linked POMs, this conductivity seems to depend on the type of 3d-transition metal as well as the POM-linkage.^55,56^ Therefore, future work will focus on advanced deposition and electrical “wiring” concepts to make full use of the molecular redox activity of each individual **{Ca**_**2**_**V**_**12**_**}**.^6,52^ These studies would also enable us to address the capacity fading observed in subsequent cycling experiments. Here, we note a fast fading up to cycle 10, which is in line with the amorphization and dissolution of the active material.^53^ Post-mortem analyses after cycling support this, and pXRD shows a complete loss of crystallinity. While energy-dispersive X-ray analysis (EDX) indicates a significantly decreased vanadium content in the post-mortem cathode, while ICP-OES shows a high content of vanadium and calcium in the dissolved Li anode.

## Conclusions

In conclusion, we herein report the synthesis and characterization of a novel, highly redox-active molecular vanadium oxide. By coordination to Ca^2+^-ions individual dodecavanadate clusters are connected to form linear chains in the solid state as well as in solution. While the Ca^2+^-ions themselves do not participate in redox-reactions, the stability of the reduced cluster is greatly improved in comparison to that of the non-functionalized parent compound, resulting in five (quasi-) reversible redox-process. Initial investigations of this “electron-sponge” material as an active material in lithium-ion batteries show promising results and will hopefully foster further investigations of this material class for electrochemical energy storage systems.

## Experimental section

### Synthesis and heat treatment of {Ca_2_V_12_}

(*n*Bu_4_N)_3_[H_3_V_10_O_28_]^57^ (330 mg, 0.17 mmol, 1 eq.) and CaCl_2_·6H_2_O (60 mg, 0.27 mmol, 1.6 eq.) were dissolved in *N*,*N*-dimethyl formamide (8 ml) and stirred for 8 days at 80 °C. Diffusion of acetone into the cooled reaction mixture gave dark green crystals suitable for X-ray diffraction (yield: 94 mg, 47 μmol, 28% based on V).

### Electrochemical characterization

Electrochemical tests were carried out in Swagelok-type cells *vs.* metallic lithium using 1 M LiPF_6_ in ethylene carbonate (EC)/dimethyl carbonate (DMC) (1 : 1, v/v) as the electrolyte. Electrodes were prepared by mechanical mixing of 45 wt% POM with 40 wt% carbon black and a 15 wt% poly(vinylidene difluoride) (PVDF) binder with *N*-methyl-2-pyrrolidone (NMP). The resulting homogeneous slurry was coated on Al-foil by the doctor blade technique and dried at 120 °C for 12 h under vacuum. Each working electrode (12 mm) contained about 1 mg active material. Temperature controlled galvanostatic charge–discharge experiments were carried out on an Arbin electrochemical workstation at 25 °C. Solid-state cyclic voltammetry (ssCV) was conducted using a Bio-Logic VMP-3 potentiostat at a scan rate of 0.1 mV s^−1^.

## Conflicts of interest

There are no conflicts to declare.

## Supplementary Material

SC-011-D0SC01401J-s001

SC-011-D0SC01401J-s002

## References

[cit1] Kudo A., Miseki Y. (2009). Chem. Soc. Rev..

[cit2] Schlögl R. (2011). Angew. Chem., Int. Ed..

[cit3] Müller A., Roy S. (2003). Coord. Chem. Rev..

[cit4] Long D. L., Tsunashima R., Cronin L. (2010). Angew. Chem., Int. Ed..

[cit5] Wang Q., Ohare D. (2012). Chem. Rev..

[cit6] Ji Y., Huang L., Hu J., Streb C., Song Y. (2015). Energy Environ. Sci..

[cit7] Chen H. Y., Friedl J., Pan C. J., Haider A., Al-Oweini R., Cheah Y. L., Lin M. H., Kortz U., Hwang B. J., Srinivasan M., Stimming U. (2017). Phys. Chem. Chem. Phys..

[cit8] Sartorel A., Carraro M., Toma F. M., Prato M., Bonchio M. (2012). Energy Environ. Sci..

[cit9] Lv H., Geletii Y. V., Zhao C., Vickers J. W., Zhu G., Luo Z., Song J., Lian T., Musaev D. G., Hill C. L. (2012). Chem. Soc. Rev..

[cit10] Gao D., Trentin I., Schwiedrzik L., González L., Streb C. (2020). Molecules.

[cit11] Yin Q., Tan J. M., Besson C., V Geletii Y., Musaev D. G., Kuznetsov A. E., Luo Z., Hardcastle K. I., Hill C. L. (2010). Science.

[cit12] Streb C. (2012). Dalton Trans..

[cit13] Rausch B., Symes M. D., Chisholm G., Cronin L. (2014). Sci.

[cit14] Dalla Francesca K., Lenfant S., Laurans M., Volatron F., Izzet G., Humblot V., Methivier C., Guerin D., Proust A., Vuillaume D. (2019). Nanoscale.

[cit15] Stuckart M., Monakhov K. Y. (2019). Chem. Sci..

[cit16] Ueda T. (2018). ChemElectroChem.

[cit17] Sadakane M., Steckhan E. (1998). Chem. Rev..

[cit18] Chen J.-J. J., Barteau M. A. (2016). Ind. Eng. Chem. Res..

[cit19] Streb C., Structure and Bonding in Molecular Vanadium Oxides: From Templates *via* Host–Guest Chemistry to Applications, in Polyoxometalate-Based Assemblies and Functional Materials, ed. Y.-F. Song, Springer-Verlag, Cham, 2017, vol. 176, pp. 1–17

[cit20] Hayashi Y. (2011). Coord. Chem. Rev..

[cit21] Klemperer W. G., Marquart T. A., Yaghi O. M. (1992). Angew. Chem., Int. Ed. Engl..

[cit22] Monakhov K. Y., Bensch W., Kogerler P. (2015). Chem. Soc. Rev..

[cit23] Kastner K., Margraf J. T., Clark T., Streb C. (2014). Chem.–Eur. J..

[cit24] Li F., Carpenter S. H., Higgins R. F., Hitt M. G., Brennessel W. W., Ferrier M. G., Cary S. K., Lezama-Pacheco J. S., Wright J. T., Stein B. W., Shores M. P., Neidig M. L., Kozimor S. A., Matson E. M. (2017). Inorg. Chem..

[cit25] Liu J., Chen Z., Chen S., Zhang B., Wang J., Wang H., Tian B., Chen M., Fan X., Huang Y., Sum T. C., Lin J., Shen Z. X. (2017). ACS Nano.

[cit26] VanGelder L. E., Matson E. M. (2018). J. Mater. Chem. A.

[cit27] Kastner K., Forster J., Ida H., Newton G. N., Oshio H., Streb C. (2015). Chem.–Eur. J..

[cit28] VanGelder L. E., Petel B. E., Nachtigall O., Martinez G., Brennessel W. W., Matson E. M. (2018). ChemSusChem.

[cit29] Schreiber E., Hartley N. A., Brennessel W. W., Cook T. R., McKone J. R., Matson E. M. (2019). ACS Appl. Energy Mater..

[cit30] Hartung S., Bucher N., Chen H.-Y., Al-Oweini R., Sreejith S., Borah P., Yanli Z., Kortz U., Stimming U., Hoster H. E., Srinivasan M. (2015). J. Power Sources.

[cit31] Schwarz B., Forster J., Anjass M. H., Daboss S., Kranz C., Streb C. (2017). Chem. Commun..

[cit32] Schwarz B., Forster J., Goetz M. K., Yücel D., Berger C., Jacob T., Streb C. (2016). Angew. Chem., Int. Ed..

[cit33] Vangelder L. E., Kosswattaarachchi A. M., Forrestel P. L., Cook T. R., Matson E. M. (2018). Chem. Sci..

[cit34] Lu S., Lv Y., Ma W., Lei X., Zhang R., Liu H., Liu X. (2017). Inorg. Chem. Front..

[cit35] Chen J.-J., Symes M. D., Fan S.-C., Zheng M.-S., Miras H. N., Dong Q.-F., Cronin L. (2015). Adv. Mater..

[cit36] Greiner S., Anjass M. H., Fichtner M., Streb C. (2020). Inorg. Chem. Front..

[cit37] Petel B. E., Brennessel W. W., Matson E. M. (2018). J. Am. Chem. Soc..

[cit38] Anjass M. H., Kastner K., Nägele F., Ringenberg M., Boas J. F., Zhang J., Bond A. M., Jacob T., Streb C. (2017). Angew. Chem., Int. Ed..

[cit39] Kastner K., Lechner M., Weber S., Streb C. (2017). ChemistrySelect.

[cit40] Tucher J., Peuntinger K., Margraf J. T., Clark T., Guldi D. M., Streb C. (2015). Chem.–Eur. J..

[cit41] Seliverstov A., Streb C. (2014). Chem.–Eur. J..

[cit42] Anjass M. H., Kastner K., Florian N., Ringenberg M., Boas J. F., Zhang J., Bond A. M., Jacob T., Streb C. (2017). Angew. Chem., Int. Ed..

[cit43] Schwarz B., Dürr M., Kastner K., Heber N., Ivanović-Burmazović I., Streb C. (2019). Inorg. Chem..

[cit44] Müller A., Das S. K., Kögerler P., Bögge H., Schmidtmann M., Trautwein A. X., Schünemann V., Krickemeyer E., Preetz W. (2000). Angew. Chem., Int. Ed..

[cit45] Demeter M., Neumann M., Reichelt W. (2000). Surf. Sci..

[cit46] Silversmit G., Depla D., Poelman H., Marin G. B., De Gryse R. (2004). J. Electron Spectrosc. Relat. Phenom..

[cit47] Hryha E., Rutqvist E., Nyborg L. (2011). Surf. Interface Anal..

[cit48] Nambu J., Ueda T., Guo S.-X., Boas J. F., Bond A. M. (2010). Dalton Trans..

[cit49] Bard A. J. and Faulkner L. R., Electrochemical Methods: Fundamentals and Applications, John Wiley & Sons, 2nd edn, 2001

[cit50] Gómez-Gil J. M., Laborda E., Gonzalez J., Molina A., Compton R. G. (2017). J. Phys. Chem. C.

[cit51] Anjass M. H., Deisböck M., Greiner S., Fichtner M., Streb C. (2018). ChemElectroChem.

[cit52] Wang H., Isobe J., Matsumura D., Yoshikawa H. (2018). J. Solid State Electrochem..

[cit53] Uematsu S., Quan Z., Suganuma Y., Sonoyama N. (2012). J. Power Sources.

[cit54] Ni E., Tsukada T., Wen Q., Sonoyama N. (2019). J. Electrochem. Soc..

[cit55] Turo M. J., Chen L., Moore C. E., Schimpf A. M. (2019). J. Am. Chem. Soc..

[cit56] Chen L., San K. A., Turo M. J., Gembicky M., Fereidouni S., Kalaj M., Schimpf A. M. (2019). J. Am. Chem. Soc..

[cit57] Klemperer W. G., in Inorganic Syntheses, John Wiley & Sons, 1990, vol. 27, p. 83

[cit58] Dolomanov O. V., Bourhis L. J., Gildea R. J., Howard J. A. K., Puschmann H. (2009). J. Appl. Crystallogr..

