# Supplementary material for: Redox-inactive ions control the redox-activity of molecular vanadium oxides
Source: Chem Sci. 2020 Apr 6;11(17):4450–5. doi: 10.1039/d0sc01401j (PMC8159454; doi:10.1039/d0sc01401j)
Supplement: SC-011-D0SC01401J-s001 [file SC-011-D0SC01401J-s001.pdf]

Electronic Supplementary Material (ESI) for Chemical Science.
This journal is © The Royal Society of Chemistry 2020

Electronic Supporting Information

Redox-inactive ions control the redox-activity of molecular vanadium oxides

Simon Greiner‡, Benjamin Schwarz‡, Mark Ringenberg, Ivana Ivanovic-Burmazovic, Maximilian Fichtner, Montaha H. Anjass*and Carsten Streb*

1. Instrumentation

Single-crystal X-ray diffraction (scXRD) was measured on a Bruker APEX-II CCD Single-crystal X-ray diffractometer equipped with a graphite monochromator using $Mo_{K\alpha}$ radiation (wavelength $\lambda(Mo_{K\alpha})$ = 0.71073 Å).

Powder X-ray diffraction (pXRD) was collected using a STOE Stadi P diffractometer with $Cu_{K\alpha}$ (λ = 1.5406 Å) radiation in the range between 3 ° to 90 °.

Attenuated total reflectance-fourier-transformed infrared spectroscopy (ATR-FT-IR) was recorded using a PerkinElmer Spectrum Two spectrometer in a range between 4000 and 500 cm^{-1}.

UV-vis spectroscopy was performed on a Varian Cary 50 spectrophotometer in a standard cuvette (d = 10.0 mm)

Inductively coupled plasma optical emission spectroscopy (ICP-OES) was performed on a Spectro Arcos FHS12.

Thermogravimetric analysis (TGA) was carried out on a NETZSCH TG 209F1 analyser at a heating rate of 10.0 K min^{-1} in a range between 30 and 700 °C under air in an Al_2O_3 crucible.

EPR spectroscopy was performed at X-band frequencies (~ 9.5 GHz) on DMF solutions (concentration: 1.0 mM) frozen to 100 K. The spectra were recorded using a Bruker System EMX with a liquid nitrogen finger dewar inserted into the standard TE_{102} rectangular cavity.

Mass spectrometry: Electrospray-ionization mass spectrometry (ESI-MS) was performed using *ultra-high resolution* time-of-flight (UHR-TOF) Bruker Daltonik maXis mass spectrometer in negative ion detection mode. Measurement conditions: source voltage: 4 kV, sample flow rate: 500 µl/h, drying gas temperature (N2): 180 °C. Before each series of measurements, the spectrometer was calibrated with Agilent "ESI-TOF low concentration tuning mixture". For cryospray-ionization mass spectrometric measurements, the UHR-TOF Bruker Daltonik maXis mass spectrometer was coupled to a Bruker cryospray unit. The drying gas (N2) was held at -55 °C and the spray gas was held at -60 °C.

X-ray photoelectron spectroscopy (XPS) was performed on Physical Electronics PHI 5800 spectrometer using monochromatized $Al_{K\alpha}$ (1486.6 eV) radiation. The measurements were performed with a detection angle of 45 °C, using pass energies at the analyzer of 93.9 and 29.35 eV for survey and detail spectra, respectively. The samples were neutralized with electrons from a flood gun (current 3 µA) to compensate for charging effects at the surface.

2. Electrochemical characterization

Solution electrochemistry: DC cyclic voltammetry (CV) experiments were performed on a Pine Research WaveDriver electrochemical workstation equipped with a standard three-electrode arrangement: working electrode: glassy carbon electrode (d = 3.0 mm), quasi reference electrode: Ag wire (in a glass frit containing electrolyte solution), counter electrode: Pt wire. All potentials are quoted relative to the ferrocene/ferrocenium internal standard.
Bulk electrolysis (BE) was conducted using a glassy carbon tube working electrode, which was placed in a two-compartment U-tube. Counter electrode Pt wire was separated by a porous glass frit and the

reference electrode was an Ag wire placed in a glass frit containing electrolyte solution. During the experiment the working electrode was kept at the appropriate potential while the solution was stirred vigorously. The electrolysis was stopped once the current dropped below 1% of the initial current.
All experiments were performed in dry DMF using $n$$Bu_4NPF_6$ (0.1 M) as supporting electrolyte. The solutions were purged with argon for at least 15 min to remove O_2 and kept under a slight positive Ar pressure while performing the experiments.

Battery performance: Electrochemical tests were carried out in Swagelok-type cells vs. metallic lithium using 1 M $LiPF_6$ in ethylene carbonate (EC)/dimethyl carbonate (DMC) (1:1, v/v) as electrolyte. Electrodes were prepared by mechanical mixing of 45 wt.-% POM with 40 wt.-% carbon black (Super P®) and 15 wt.-% poly(vinylidene difluoride) (PVDF) binder with *N*-methyl-2-pyrrolidone (NMP). The resulting homogeneous slurry was coated on Al-foil by doctor blade technique and dried at 120 °C for 12 h under vacuum. Each working electrode (12 mm) contained about 1 mg active material. Temperature controlled galvanostatic charge-discharge experiments were measured in an Arbin electrochemical workstation at 25 °C. Solid-state cyclic voltammetry (ssCV) was conducted using a Bio-Logic VMP-3 potentiostat at a scan rate of 0.1 mV s^{-1}.

3. Experimental procedure

All chemicals were purchased from Sigma Aldrich, VWR or Alfa Aesar and were of reagent grade. The chemicals were used without further purification unless stated otherwise.

Synthesis and heat treatment of ($n$$Bu_4N)_2[Ca_2V_{12}O_{32}Cl(DMF)_3]$ x (DMF) ({Ca_2V_{12}}):

($n$$Bu_4N)_3[H_3V_{10}O_{28}]$, as prepared according to literature[1], (330 mg, 0.2 mmol, 1 eq.) and $CaCl_2$ x 6 H_2O (60 mg, 0.27 mmol, 1.6 eq.) were dissolved in *N,N*-dimethyl formamide (8 ml) and stirred for 8 days at 80 °C. Diffusion of acetone into the cooled reaction mixture yielded dark green crystals suitable for X-ray diffraction.
Yield: 94 mg (47 µmol, 28% based on V).
Characteristic IR bands (in cm^{-1}): 2960 (w), 2932 (w), 2871 (w), 1646 (s), 1482 (m), 1462 (m), 1377 (m), 1253 (w), 1117 (w), 1063 (w), 987 (vs), 879 (w), 817 (s), 757 (s), 651 (s), 607 (vs).
UV/vis (in DMF): ε_{345} = 15644 M^{-1} cm^{-1}, ε_{978} = 1118 M^{-1} cm^{-1}.
ICP-OES in wt.-% (calcd.): Ca 4.16 (4.12), V 31.67 (31.48), Ca:V atomic ratio is 1:5.98.
Elemental analysis in wt.-% (calcd.): C 26.42 (26.21), H 5.19 (5.00), N 4.08 (4.17).
{Ca_2V_{12}}-120 was obtained by heating **{Ca_2V_{12}}** for 12 h at 120 °C under vacuum.

4. Crystallographic section

Suitable single-crystals of **{Ca_2V_{12}}** were mounted onto a microloop using Fomblin oil. X-ray diffraction intensity data were measured at 150 K on a Agilent SuperNova or on a Bruker D8 QUEST diffractometer λ($Mo_{K\alpha}$ = 0.71073 Å) equipped with a graphite monochromator. Structure solution was carried out using SHELX-2013[2] package through OLEX2[3] Corrections for incident and diffracted beam absorption effects were applied using empirical methods.[4] Structures were solved by a combination of direct methods and difference Fourier syntheses and refined against F^2 by the full matrix least-squares technique. Non-hydrogen atoms were refined anisotropically. Hydrogen atoms were added to carbon atoms using a riding model. The geometry and anisotropic refinement of the solvent and ligand molecules was restrained using SIMU and DELU.
The metal oxo framework was refined fully anisotropically and no restraints were used. CIF file can be obtained free of charge from the CCDC.

Table S1 Crystallographic parameters for **{Ca_2V_{12}}**.

Compound code	**{Ca_2V_{12}}**
CCDC code	1983287
Empirical formula	$C_{44}H_{96}N_6O_{36}ClV_{12}Ca_2$
Formula weight / g mol^{-1}	2012.15
Temperature / K	150
Wavelength / nm	0.71073
Crystal system	Monoclinic
Space group	$P2_1/c$
Unit cell dimensions / Å	a = 10.6954(5)<br>b = 29.2218(14)<br>c = 28.2691 (15)
Unit cell angles / °	α = 90<br>β = 95.3952<br>γ = 90
Volume / Å^3	8796.0(8)
Z	4
Density (calcd.) / g cm^{-3}	1.519
Absorption coefficient μ / mm^{-1}	1.433
F(000)	4092.0
2Θ range for data collection / °	4.198 to 52.796
Index ranges	$-13 \le h \le 13$, $-36 \le k \le 36$, $-35 \le l \le 34$
No. reflections	204730
Independent reflections	18025 [R_{int} = 0.0727, R_{sigma} = 0.0308]
Data / restraints / parameters	18025/462/974
Goodness-of-fit	1.165
Final R indices [I>2sigma(I)]	R_1 = 0.0815, wR_2 = 0.1795
R indices (all data)	R_1 = 0.0960, wR_2 = 0.1883
Largest diff. peak and hole	1.28/-0.99

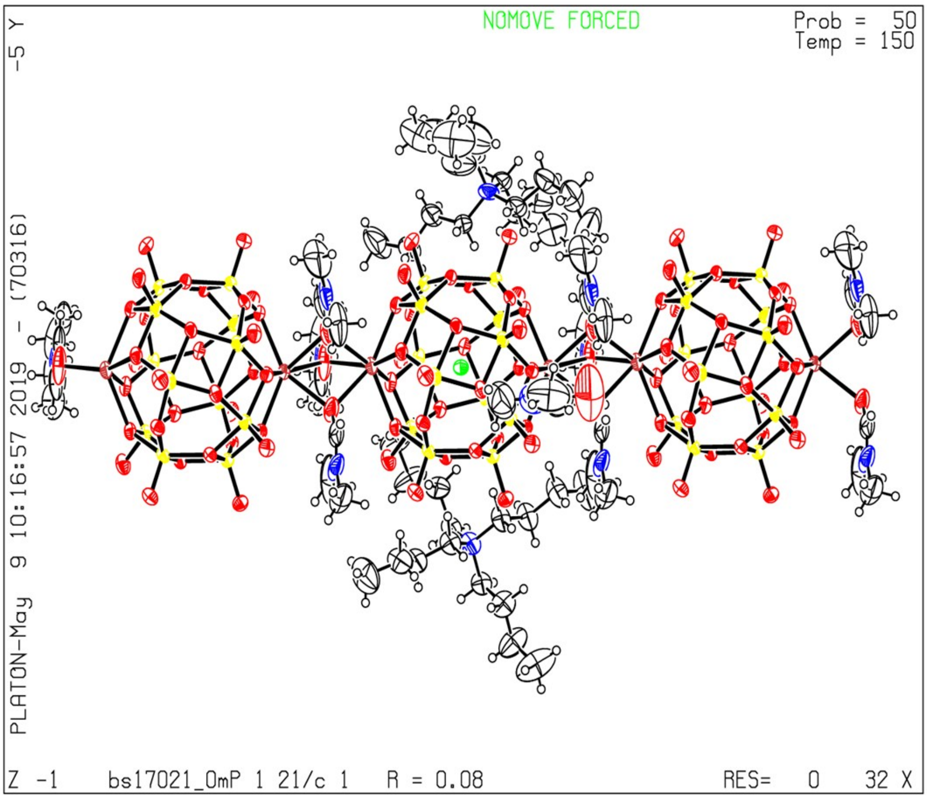

Figure S 1 ORTEP-representation of **{Ca_2V_{12}}**, probability ellipsoids shown at 50%.

5. X-ray photoelectron spectroscopy

Figure S 2 shows XPS measurements and fitting of **{Ca_2V_{12}}**. From the fitting of the V 2p spectrum, the obtained binding energy values of **{Ca_2V_{12}}** are for V $2p_{3/2}$ 517.2 eV, V $2p_{1/2}$ 524.5 eV and a V $2p_{3/2}$ – V $2p_{1/2}$ splitting with 7.3 eV confirming mainly V^{V} oxidation state.[5,6] The presence of V^{IV} is indicated by shoulder around 515.7 eV combined with a peak broadening of the V^{5+} $2p_{3/2}$ peak leading to an asymmetric peak shape.[7] Due to the asymmetric peak shape no quantification of the ratio between V^{V} and V^{IV} was attempted.

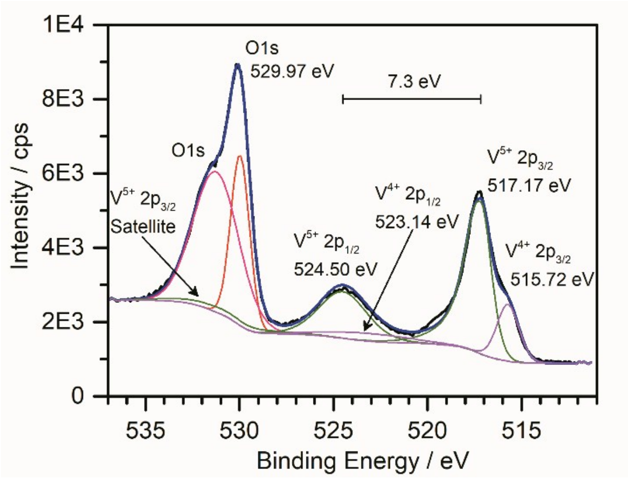

Figure S 2 XPS spectrum of as-prepared **{Ca_2V_{12}}**.

6. Electron paramagnetic resonance spectroscopy

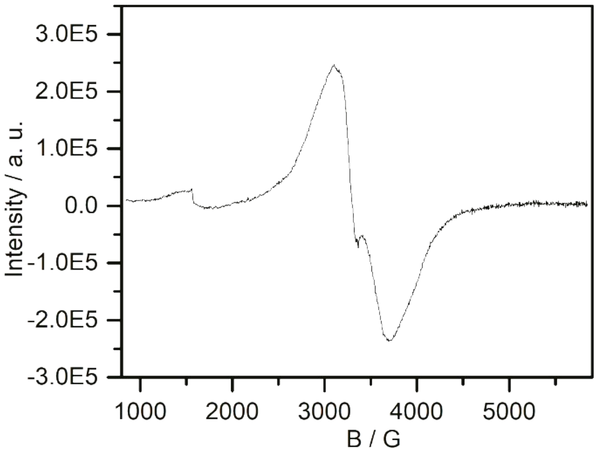

Figure S 3 EPR spectrum of as-prepared **{Ca_2V_{12}}** (1 mM in frozen DMF solution (containing 0.1 M *(n*Bu_4N*)*PF_6) at 77 K.

7. Mass spectrometry of {Ca_2V_{12}}

Table S2 Calcium vanadate species observed in negative mode mass spectra.

Found m/z	Simulated m/z	Charge	Assignment
387.710	387.710	3-	$[CaV^{IV}V^{V}_{11}O_{32}]^{3-}$
399.703	399.702	3-	$\{H[Ca_2V^{IV}_3V^{V}_9O_{32}Cl]\}^{3-}$
412.687	412.687	3-	$[Ca_2V^{IV}_2V^{V}_{10}O_{32}Cl]^{3-}$
619.030	619.029	2-	$[Ca_2V^{IV}V^{V}_{11}O_{32}Cl]^{2-}$
655.556	655.555	2-	$\{[Ca_2V^{IV}V^{V}_{11}O_{32}Cl](dmf)\}^{2-}$
663.554	663.553	2-	$\{[Ca_2V_{12}O_{32}Cl](dmf)(O)\}^{2-}$
692.083	692.082	2-	$\{[Ca_2V^{IV}V^{V}_{11}O_{32}Cl](dmf)_2\}^{2-}$
906.134	906.135	3-	$\{(nBu_4N)[Ca_2V^{IV}V^{V}_{11}O_{32}Cl]_2\}^{3-}$
930.486	930.486	3-	$\{(nBu_4N)[Ca_2V^{IV}V^{V}_{11}O_{32}Cl]_2(dmf)\}^{3-}$
954.834	954.837	3-	$\{(nBu_4N) [Ca_2V^{IV}V^{V}_{11}O_{32}Cl]_2(dmf)_2\}^{3-}$
1516.867	1516.871	2-	$\{(nBu_4N)_2[Ca_2V^{IV}V^{V}_{11}O_{32}Cl]_2(dmf)\}^{2-}$
1553.389	1553.398	2-	$\{(nBu_4N)_2[Ca_2V^{IV}V^{V}_{11}O_{32}Cl]_2(dmf)_2\}^{2-}$
1626.443	1626.450	1-	$\{(nBu_4N) [Ca_2V^{IV}V^{V}_{11}O_{32}Cl](dmf)_2\}^{-}$

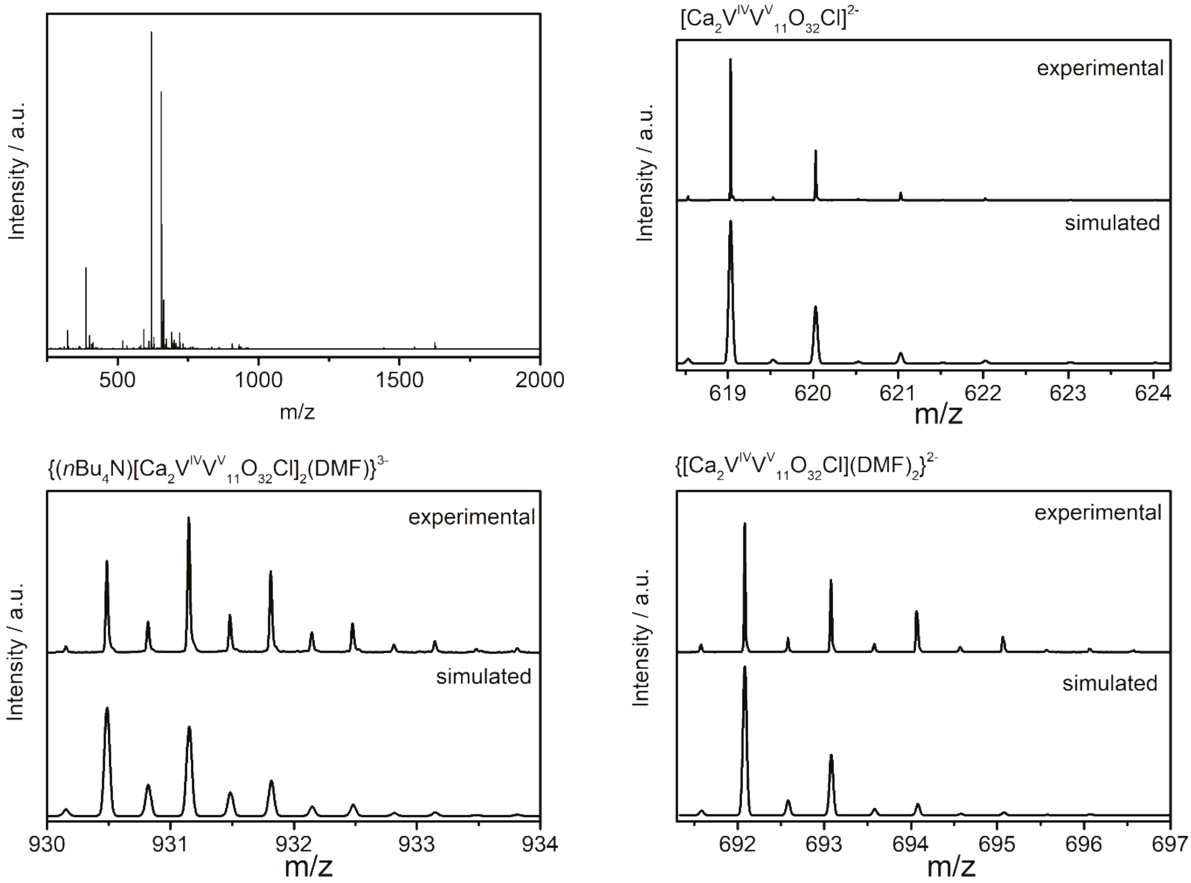

Figure S 4 Top left: High resolution electrospray ionization mass-spectrometry of **{Ca_2V_{12}}** (ca. $5x10^{-5}$ M in DMF). **Top right:** Experimental and simulated ESI mass spectrum of the full cluster anion $[Ca_2V^{IV}V^{V}_{11}O_{32}Cl]^{2-}$ observed at m/z = 619.030. **Bottom left:** Experimental and simulated spectrum of the cluster dimer $\{(nBu_4N)_2[Ca_2V^{IV}V^{V}_{11}O_{32}Cl]_2(dmf)\}^{3-}$ observed at m/z = 930.486. **Bottom right:** Experimental and simulated ESI mass spectrum of the full cluster anion $[Ca_2V^{IV}V^{V}_{11}O_{32}Cl(dmf)_2]^{2-}$ found at m/z = 692.083.

8. Solution Electrochemical analysis

a. Cyclic voltammetry of {Ca_2V_{12}} and {V_{12}}

CV of **{Ca_2V_{12}}** and **{V_{12}}** over the scan range shows five quasi-reversible redox couples at $E_{I/I'}$ = -0.31 V, $E_{II/II'}$ = -0.64 V, $E_{III/III'}$ = -1.06 V, $E_{IV/IV'}$ = -1.48 V und $E_{V/V'}$ = -1.95 V.

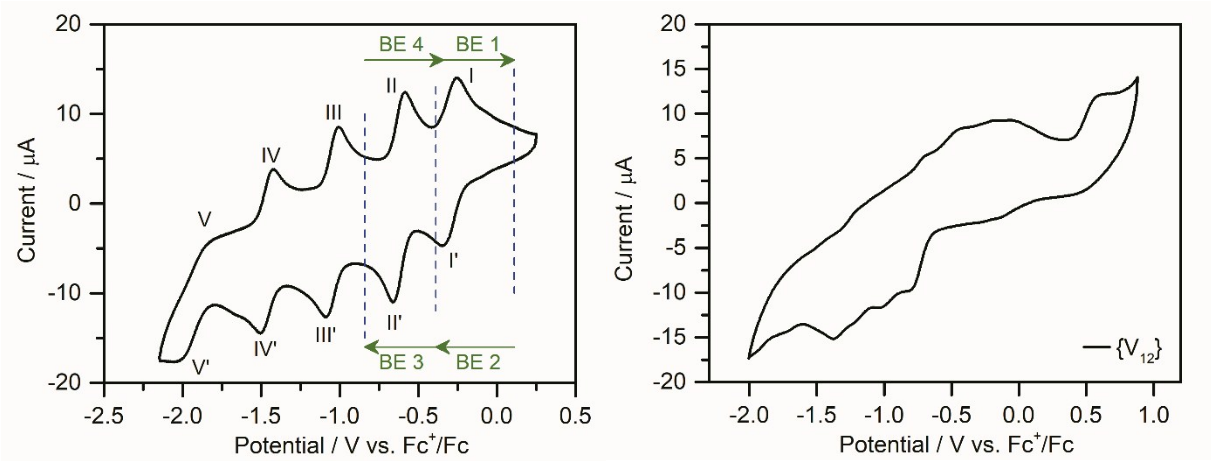

Figure S 5 Cyclic voltammogram of **{Ca_2V_{12}}** (left) and **{V_{12}}** (right) in de-aerated, anhydrous DMF containing 0.1 M (nBu$_4$N)PF$_6$ as electrolyte, [analyte] = 1 mM, scan rate = 0.1 V s^{-1}.

b. Scan rate dependent cyclic voltammetry

CV experiments at varied scan rates were performed to investigate the reversibility of process I/I' to IV/IV'.

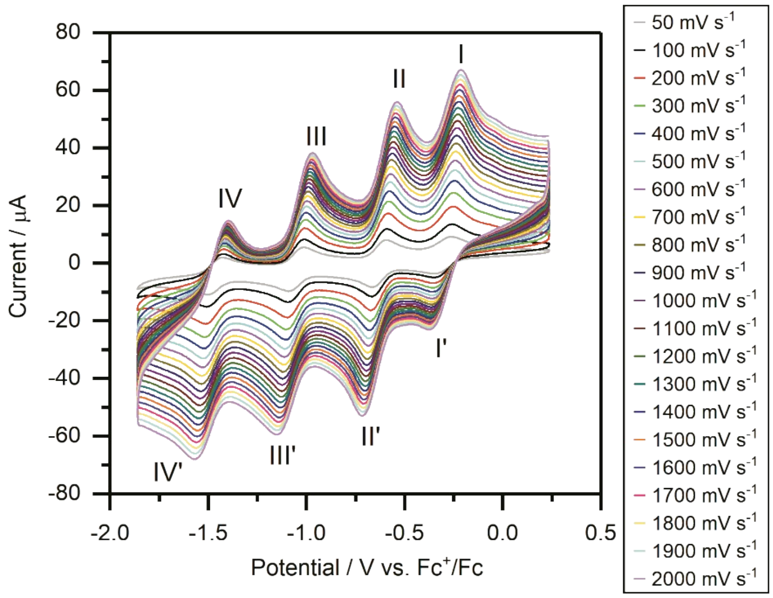

Figure S 6 Cyclic voltammogram of **{Ca_2V_{12}}** in de-aerated, anhydrous DMF containing 0.1 M (nBu$_4$N)PF$_6$ as electrolyte, [**{Ca_2V_{12}}**] = 1 mM, scan rate varied between 50 - 2000 mV s^{-1}.

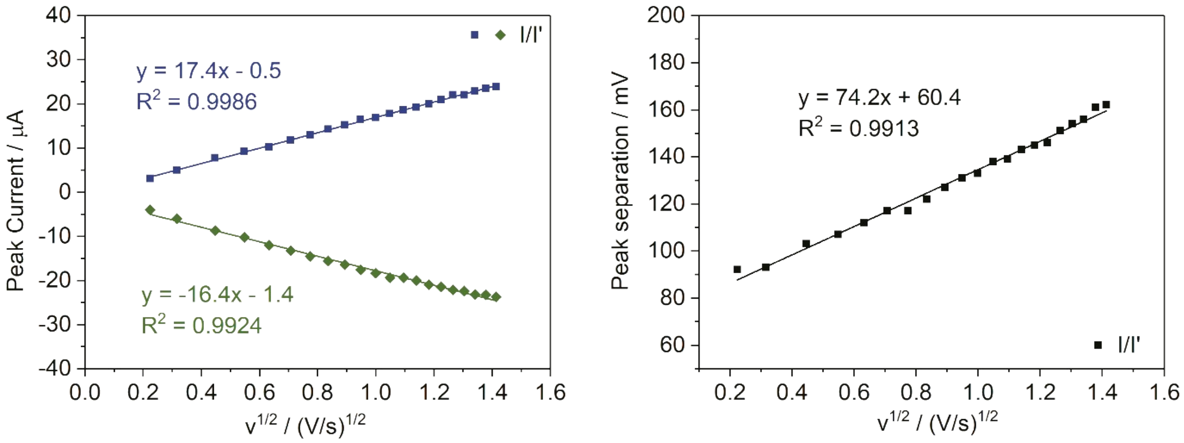

Figure S 7 Plots of i_p vs. $v^{1/2}$ (left) and ΔE vs. $v^{1/2}$ (right) for the first redox process ($E_{1/2}$ = -0.31 V) of **{Ca_2V_{12}}**.

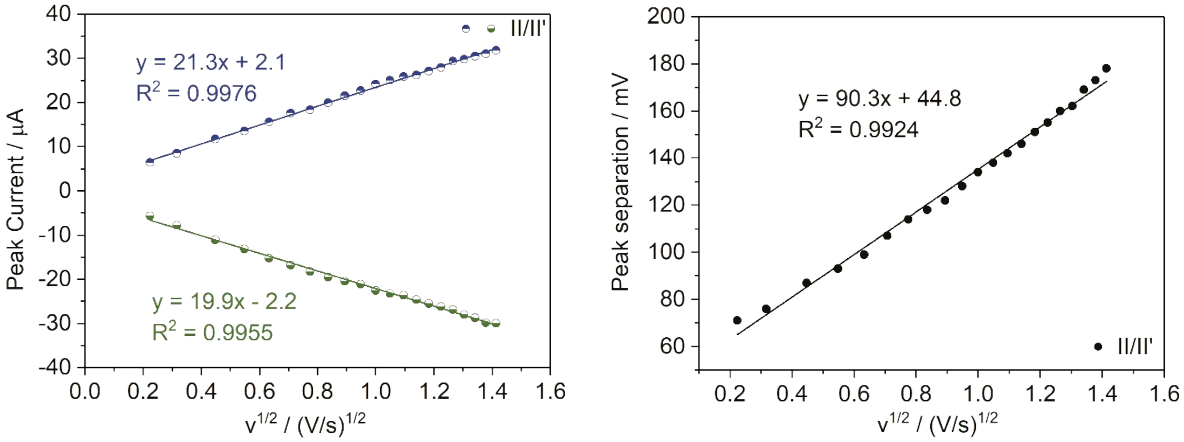

Figure S 8 Plots of i_p vs. $v^{1/2}$ (left) and ΔE vs. $v^{1/2}$ (right) for the second redox process ($E_{1/2}$ = -0.64 V) of **{Ca_2V_{12}}**.

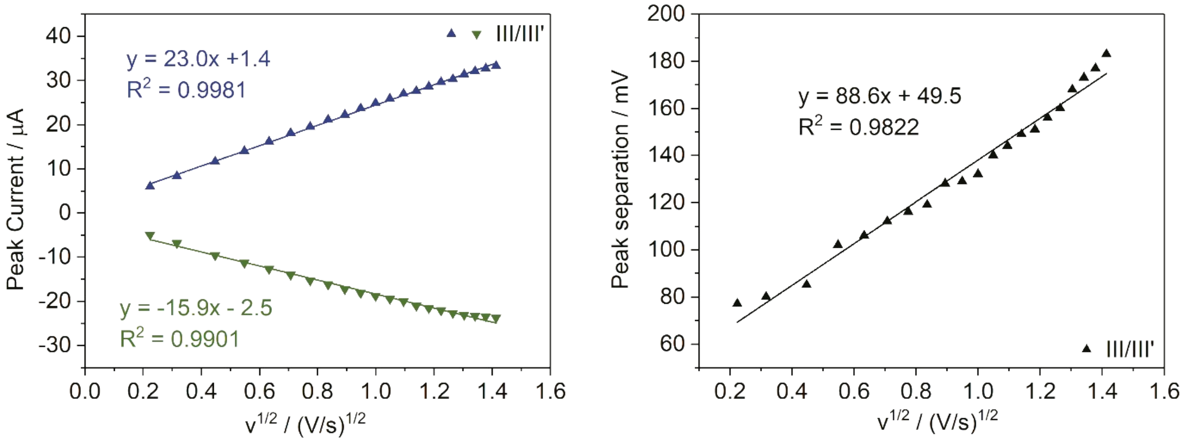

Figure S 9 Plots of i_p vs. $v^{1/2}$ (left) and ΔE vs. $v^{1/2}$ (right) for the third redox process ($E_{1/2}$ = -1.06 V) of **{Ca_2V_{12}}**.

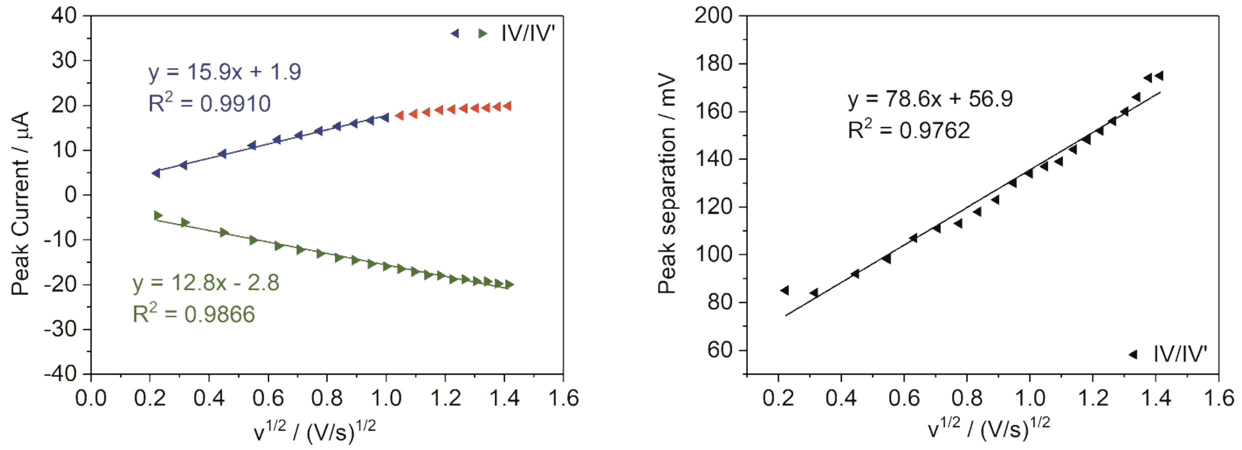

Figure S 10 Plots of i_p vs. $v^{1/2}$ (left) and ΔE vs. $v^{1/2}$ (right) for the fourth redox process ($E_{1/2}$ = -1.48 V) of **{Ca_2V_{12}}**.

Table S3 Ratio of peak currents observed in cyclic voltammetry.

Process	$\lvert i_{p, ox}\rvert / \lvert i_{p, red}\rvert$ @ 0.1 V s^{-1}
I/I’	0.83
II/II’	1.09
III/III’	1.21
IV/IV’	1.08

c. Cyclic voltammetry in the presence of Li^+ salts

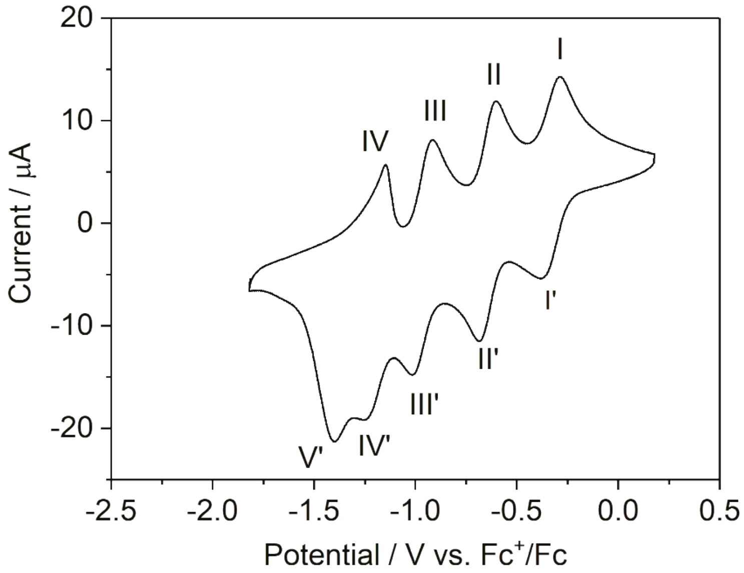

Figure S 11 Cyclic voltammogram of **{Ca_2V_{12}}** in de-aerated, anhydrous DMF containing 0.1 M $LiPF_6$ as electrolyte, [**{Ca_2V_{12}}**] = 1 mM, scan rate = 0.1 V s^{-1}.

d. Spectro-Electrochemistry of {Ca_2V_{12}}

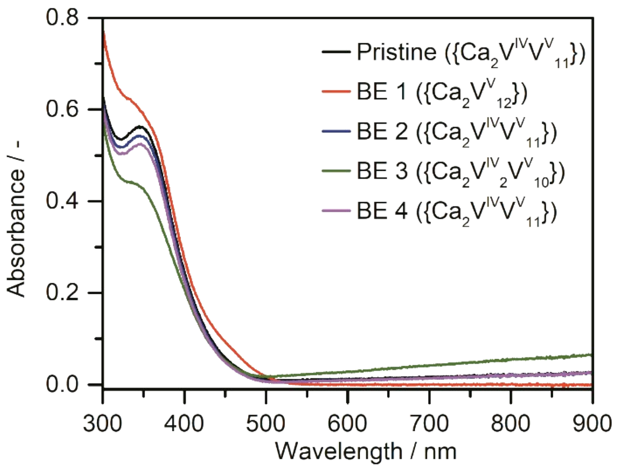

Figure S 12 UV-vis-NIR absorption spectra of pristine **{Ca_2V_{12}}** (black); after full oxidation (BE 1) (at E = 0.11 V, red), after re-reduction to the native state (BE2) (at E = -0.39 V, blue), after reduction (BE 3) (at E = -0.84 V, green) and re-oxidation to the native state (BE 4) (at E = -0.34 V, magenta). All spectra were recorded in DMF. For the fully oxidized **{Ca_2V_{12}}**, no absorbance is observed in the NIR region. All potentials are reported vs. Fc^+/Fc.

Table S4 Electrons transferred and change in the IVCT intensity upon bulk electrolysis.

**Reaction**		**e^- transferred**	**ε_{900} / M^{-1} cm^{-1}**
Oxidation (0.11 V)	**BE 1**	-1.04	0
First Reduction (-0.39 V)	**BE 2**	+0.96	687
Second Reduction (-0.84 V)	**BE 3**	+1.12	1828
Re-oxidation (-0.34 V)	**BE 4**	-1.01	714
**In total**		**+2.08 / -2.05**	

9. Thermogravimetric analysis (TGA)

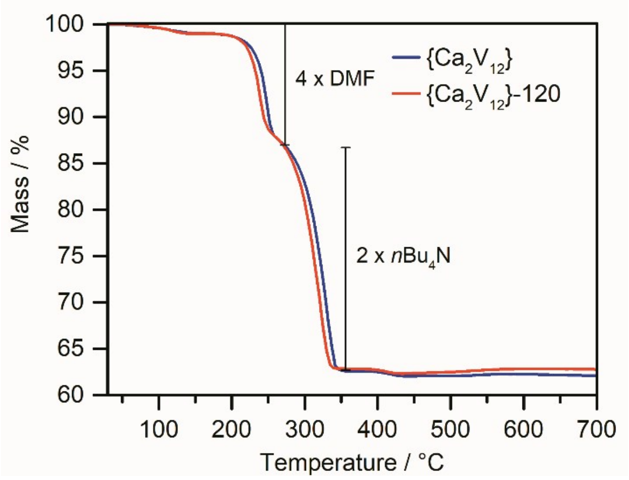

Figure S 13: Thermogravimetric analysis (under air) of **{Ca_2V_{12}}** before (blue) and after heating to 120 °C under vacuum for 12 h (red) indicating a weight loss corresponding to the loss of four DMF (25 - 275 °C) and two (*n*Bu_4N) molecules (275 – 350 °C) (observed: 37.2 wt.-%; calcd.: 38.5 wt.-%). Identical thermal behavior is observed for both samples.

10. Thermal stability

e. Infrared spectroscopy

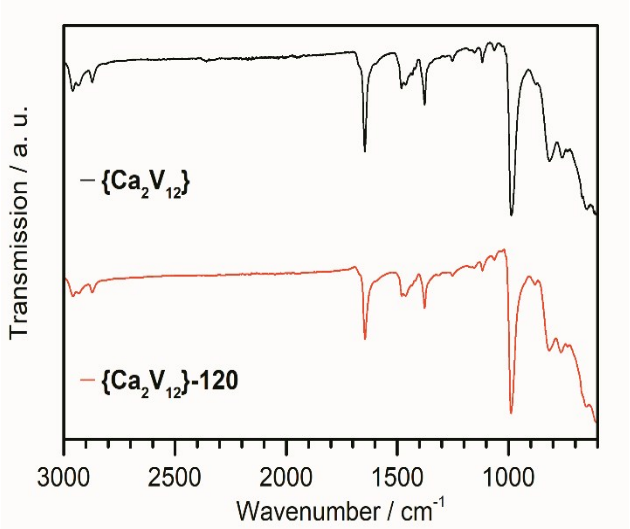

Figure S 14 ATR-FT-IR spectra of **{Ca_2V_{12}}** (black) and after heating under electrode fabrication conditions (12 h at 120 °C under vacuum; **{Ca_2V_{12}} -120** (red)).

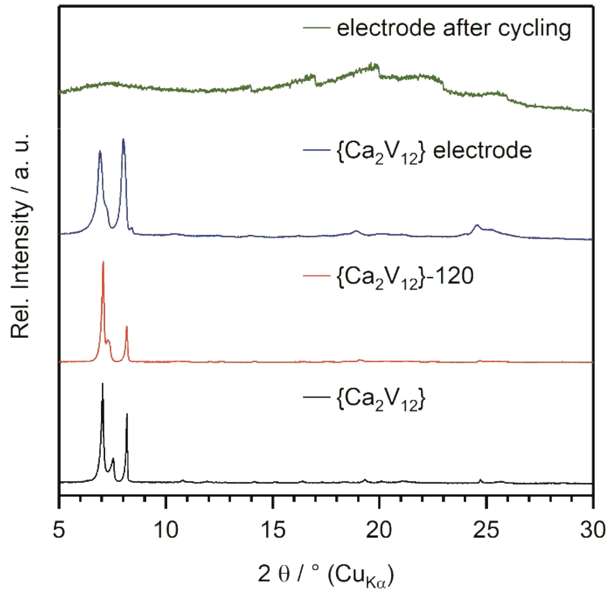

Figure S 15 PXRD pattern (Cu K_{α}) of as-prepared **{Ca_2V_{12}}** (black), after heating under electrode fabrication conditions (**{Ca_2V_{12}} -120**; red), as-prepared electrode used for battery tests (blue) and the electrode after cycling between 2.0 and 3.8 V vs. Li^+/Li (green).

f. Powder X-ray Diffraction

11. Battery performance

a. Solid-state cyclic voltammetry

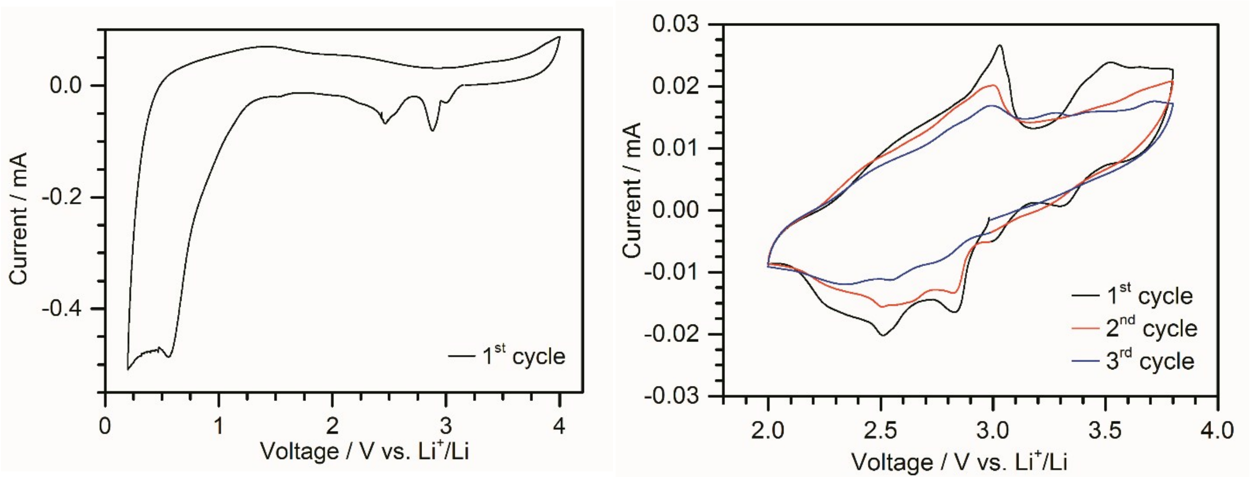

Figure S 16 Left: Cyclic voltammogram (CV) of **{Ca_2V_{12}}** coated on a stainless-steel current collector containing 40 wt.-% carbon black and 15 wt.-% PVDF. Conditions: 0.2 – 4.0 V vs. Li^+/Li; scan rate: 0.2 mV s^{-1}. **Right:** CV of as-prepared electrodes in a voltage range of 2.0 – 3.8 V vs. Li^+/Li at a scan rate of 0.2 mV s^{-1}.

b. Galvanostatic testing of {Ca_2V_{12}}

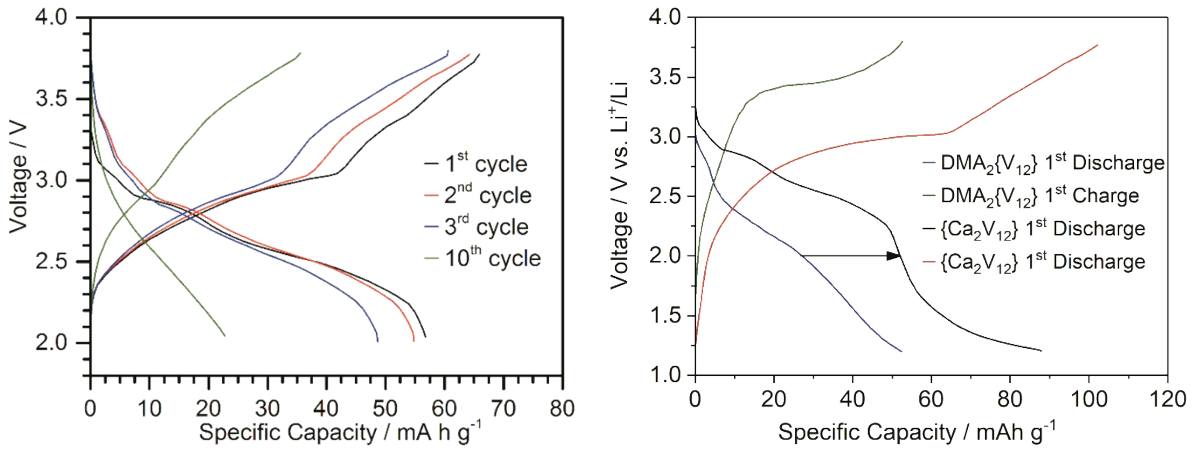

Figure S 17 Left: Galvanostatic charge-discharge profile of **{Ca_2V_{12}}** in the voltage range of 2.0 – 3.8 V versus Li^+/Li at a current density of 50 mA g^{-1}. **Right:** Comparison of the galvanostatic charge-discharge profiles of **{Ca_2V_{12}}** and **{V_{12}}** in the voltage range of 1.2 – 3.8 V versus Li^+/Li at a current density of 50 mA g^{-1}.

12. References

[1] Walter G. Klemperer, in *Inorg. Synth. Vol. 27*, John Wiley & Sons, **1990**, p. 83.

[2] George M. Sheldrick, *Acta Crystallogr. Sect. C* **2015**, *71*, 3–8.

[3] O. V. Dolomanov, L. J. Bourhis, R. J. Gildea, J. A. K. Howard, H. Puschmann, *J. Appl. Crystallogr.* **2009**, *42*, 339–341.

[4] B. Y. R. H. Blessing, *Acta Crystallogr. Sect. A* **1995**, *51*, 33–38.

[5] G. Silversmit, D. Depla, H. Poelman, G. B. Marin, R. De Gryse, *J. Electron Spectros. Relat. Phenomena* **2004**, *135*, 167–175.

[6] E. Hryha, E. Rutqvist, L. Nyborg, *Surf. Interface Anal.* **2012**, *44*, 1022–1025.

[7] M. Demeter, M. Neumann, W. Reichelt, *Surf. Sci.* **2000**, *454*, 41–44.

13. Author Contributions

All authors conceived the experiments. S.G. and B.S. performed synthesis, spectroscopy and data analysis. S. G. and B.S. performed electrochemistry. B.S. performed spectro-electrochemistry. S.G. and M.H.A. performed battery tests. M.R. performed EPR spectroscopy. M.D. and I.I.B. performed Mass spectrometry. S.G., B.S., M.H.A. and C.S. co-wrote the manuscript. S.G. and B.S. contributed equally to this study.
